# Increased liver-specific proteins in circulating extracellular vesicles as potential biomarkers for drug- and alcohol-induced liver injury

**DOI:** 10.1371/journal.pone.0172463

**Published:** 2017-02-22

**Authors:** Young-Eun Cho, Eun-Ju Im, Pyong-Gon Moon, Esteban Mezey, Byoung-Joon Song, Moon-Chang Baek

**Affiliations:** 1 Department of Molecular Medicine, Cell and Matrix Research Institute, School of Medicine, Kyungpook National University, Daegu, Republic of Korea; 2 Section of Molecular Pharmacology and Toxicology, Laboratory of Membrane Biochemistry and Biophysics, National Institute on Alcohol Abuse and Alcoholism, NIH, Bethesda, Maryland, United States of America; 3 Department of Medicine, The Johns Hopkins University School of Medicine, Baltimore, Maryland, United States of America; Kyungpook National University School of Medicine, REPUBLIC OF KOREA

## Abstract

Drug- and alcohol-induced liver injury are a leading cause of liver failure and transplantation. Emerging evidence suggests that extracellular vesicles (EVs) are a source of biomarkers because they contain unique proteins reflecting the identity and tissue-specific origin of the EV proteins. This study aimed to determine whether potentially hepatotoxic agents, such as acetaminophen (APAP) and binge alcohol, can increase the amounts of circulating EVs and evaluate liver-specific EV proteins as potential biomarkers for liver injury. The circulating EVs, isolated from plasma of APAP-exposed, ethanol-fed mice, or alcoholic hepatitis patients versus normal control counterparts, were characterized by proteomics and biochemical methods. Liver specific EV proteins were analyzed by immunoblots and ELISA. The amounts of total and liver-specific proteins in circulating EVs from APAP-treated mice significantly increased in a dose- and time-dependent manner. Proteomic analysis of EVs from APAP-exposed mice revealed that the amounts of liver-specific and/or hepatotoxic proteins were increased compared to those of controls. Additionally, the increased protein amounts in EVs following APAP exposure returned to basal levels when mice were treated with *N*-acetylcysteine or glutathione. Similar results of increased amounts and liver-specific proteins in circulating EVs were also observed in mice exposed to hepatotoxic doses of thioacetamide or d-galactosamine but not by non-hepatotoxic penicillin or myotoxic bupivacaine. Additionally, binge ethanol exposure significantly elevated liver-specific proteins in circulating EVs from mice and alcoholics with alcoholic hepatitis, compared to control counterparts. These results indicate that circulating EVs in drug- and alcohol-mediated hepatic injury contain liver-specific proteins that could serve as specific biomarkers for hepatotoxicity.

## Introduction

Extracellular vesicles (EVs), including exosomes (40–150 nm in diameter) and microvesicles (50–1,000 nm in diameter), are constantly released by most types of cells[1–3]. EVs contain cell-type-specific proteins, mRNA, and miRNA[4] and provide intercellular state information in various pathogenic processes. These informative EVs have been shown to be useful biomarkers for various diseases, including cancers[5]. Moreover, recent reports have showed that exosome-associated liver-specific mRNA[6] and circulating exosomal miRNAs[7] are biomarkers for liver injury, and that exosomal proteins can also be potential biomarkers for liver injury[8]. In addition, the important roles of microvesicles as promising biomarkers in liver diseases have been reported[9].

Drug-induced liver injury (DILI), in which liver function is impaired as a result of exposure to a hepatotoxic drug, represents a major challenge for clinicians, the pharmaceutical industry, and regulatory agencies worldwide, including the Food and Drug Administration (FDA)[10]. The liver is a primary site of drug toxicity because it metabolizes exogenous compounds into reactive intermediates, which can cause acute liver failure[11]. DILI is the most frequently cited reason for abandoning compounds early in development or withdrawal of the approved drugs from the market[12]. Moreover, the dosages of many drugs, most notably acetaminophen (APAP), are limited because of their potential to induce liver injury and acute failure. APAP-induced hepatotoxicity is a common consequence of APAP overdose that can cause acute liver failure and death especially in the presence of alcohol ingestion[13].

Alcoholic liver disease (ALD) is a major cause of morbidity and mortality globally, often advancing from simple steatosis to cirrhosis, hepatocellular carcinoma to death[14]. Serum aminotransferases (alanine and aspartate aminotransferases, ALT & AST, respectively) are the biomarkers used most frequently to determine liver injury, and released into circulation following loss of membrane integrity and lysis of hepatocytes. The problem, however, is that ALT/AST levels also increase after injury to other tissues, such as skeletal muscle, and that their assessments require fresh blood samples[15] because the half-life of elevated ALT is only 10 to 60 h, depending on the species[16]. In addition, liver disease can be observed without detection of elevated levels of ALT or AST in some cases, as previously reported[17–22]. Therefore, new approaches to identify better biomarkers specific for liver injury have been introduced recently. These biomarkers include gene[23], microRNA[24], and protein profiles[25], and mechanistic biomarkers involving microRNA[26] and proteins[27] from the blood of patients with clinical hepatotoxicity.

In this study, we aimed to test whether the number and/or amount of circulating EVs are increased after drug- or alcohol-induced liver injury and the secreted EVs contain liver-specific proteins. Additionally, we determined the utility of liver-specific proteins in circulating EVs as potential biomarkers for liver toxicity in animal models and human alcoholics.

## Materials and methods

### Human subjects

All individuals provided written informed consent for blood donation according to a protocol approved by the Institutional Review Board of The Johns Hopkins University Medical Institutions (89-01-19-01 and 97-02-24-03). The characteristics of the healthy controls and alcoholics are summarized in Table 1. Serum samples derived from the study subjects were used for isolation, quantification, and characterization of EV proteins.

**Table 1:** Clinical and biochemical characteristics of the study subjects.

	Control	Alcoholics
Number of People and Male/Female ratio)	9 (7/2)	14 (10/4)
Age (Years)	39.0 ± 10.2	45.9 ± 15.5
Alcohol intake (g/day)	0	120.0 ± 42.6
Aspartate aminotransferase (U/L)	17.2 ± 3.3	69.9 ± 33.4*
Bilirubin (μmol/L)	10.0 ± 5.1	10.5 ± 10.0
Prothombin assessment (INR)	14.0 ± 1.1	14.5 ± 2.8

The values are expressed as mean ± SD.

* *P* < 0.05. Control subjects were healthy, life-time non-drinkers due to their religious reasons.

### Animal studies

Mice were cared for in compliance with the protocols approved by the Institutional Animal Use and Care Committee of Kyungpook National University (2014–0083). For drug-induced liver injury, 6-week-old male BALB/c were fasted overnight, although water was provided *ad libitum*. The next day, these mice were injected with 1 x phosphate buffered saline (PBS) or a single ip dose of 300 mg/kg acetaminophen (APAP, Sigma-Aldrich, St. Louis, MO) for 24 h. In separate experiments, APAP was administered at 75, 150, or 300 mg/kg, and sacrificed after 1 and 3 h. Other mice were injected with a single dose of 200 mg/kg and 1,000 mg/kg, respectively, of thioacetamide (TAA, Sigma-Aldrich) and d-galactosamine (DGAL, Sigma-Aldrich) for 24 h. For the myotoxic injury, bupivacaine HCl (BPVC, Sigma-Aldrich) (0.4 mL of 0.5% wt/vol) dissolved in PBS, was administered via intramuscular injection once into both the right and left tibialis anterior of the mice. For the negative control, mice were treated with a single ip dose of 2,400 mg/kg of non-hepatotoxic penicillin (PCN, Sigma-Aldrich) for 24 h. To prevent drug-induced liver injury, *N*-acetylcysteine (NAC, Sigma-Aldrich) and glutathione (GSH, Sigma-Aldrich) were made fresh in 1x PBS for each experiment. NAC and GSH were administered at the doses of 100 and 200 mg/kg, respectively. For the binge ethanol exposure, male BALB/c mice were treated via oral gavage twice with 6 g ethanol/kg or saline (control) at 12 h interval and were sacrificed 1 h after the second dose[28, 29]. At the end of the treatment period, plasma was collected from each mouse and stored at -80°C. Liver from each mouse was excised and immediately snap-frozen in liquid nitrogen for further RNA analysis. A portion of the fresh liver was preserved in 10% neutral-buffered formalin for histopathological analysis.

### Plasma collection

To obtain the serum fraction, blood was collected in serum separator vacutainer tubes (BD Biosciences, Rockville, MD). The samples were then centrifuged at 10,000 rpm for 15 min at 4°C. The upper clear fraction was carefully transferred into centrifuge tubes and stored at -80°C. To separate the plasma, blood was collected in EDTA-containing microtainer tubes (BD Biosciences) and centrifuged at 4,000–5,000 rpm for 5 min at room temperature. The centrifugation step was repeated twice to minimize platelet contamination, and the plasma fraction was stored at -80°C.

### Histological analysis and serum ALT and AST measurements

To assess the liver damage induced by APAP or other agents used in this study, sections of fresh liver tissues from treated or control animals were fixed in neutral formalin. Paraffin-embedded blocks of formalin-fixed individual liver sections were cut at 5 microns, stained with hematoxylin/eosin (H/E), and observed under a light microscope (original magnification, 10X) by a pathologist. In addition, the ALT and AST levels were determined in plasma obtained from each animal using a standard end-point colorimetric assay kit (TECO Diagnostics, Anaheim, CA).

### Culture of liver cell lines

The Hep3B and HepG2 liver cell lines were obtained from the American Type Culture Collection (Manassas, VA, USA), and these cultured cells were maintained in Dulbecco’s modified Eagle medium (DMEM) (Invitrogen, Carlsbad, CA) containing 10% fetal bovine serum (FBS) and 1% antibiotic-antimycotic (Invitrogen) at 37°C under 5% CO_2_. For all studies, vesicle-depleted (VD) media were prepared by centrifuging cell-culture medium at 100,000 *g* overnight to spin down any preexisting vesicular contents. The VD medium was changed and the cells were incubated with APAP or 1x PBS, as a control treatment, in DMEM medium containing 10% FBS and 1% antibiotic-antimycotic. HepG2 cells were treated with the IC_10_ of 0.1 mM APAP, IC_30_ of 5 mM APAP, and the IC_50_ of 25 mM APAP in growth media. Hep3B cells were treated with the IC_10_ of 0.5 mM APAP, IC_30_ of 10 mM APAP, and the IC_50_ of 50 mM APAP in growth media for 24 h. Cultured cells were collected for various experiments.

In separate experiments, hepatocytes were freshly isolated from male Sprague-Dawley rats, weighing 200–250 g, using a two-step collagenase perfusion procedure, as previously described by our group[30]. Hepatocyte viability, as assessed by trypan blue exclusion, exceeded 90%. Freshly harvested rat hepatocytes were suspended in William’s E medium containing 5% FBS, 100 units/mL penicillin, and 100 mg/mL streptomycin and were then inoculated in rat tail collagen-coated petri dishes or 6-well culture plates. Primary hepatocytes were incubated in a humidified incubator under 95% air and 5% CO_2_ at 37°C for 24 h. After reaching confluence, the rat primary hepatocytes were treated with 10 mM APAP (IC_30_) or 20 mM APAP (IC_50_) prepared in culture media.

### EV isolation from cell culture supernatant

For EV isolation from different sources of HepG2 cells, Hep3B, rat primary hepatocytes, cell culture supernatants were differentially centrifuged, as previously described, with some modifications[5, 31]. The cell culture supernatants were subjected to serial centrifugations for 3 min at 300 *g*, 20 min at 2,500 *g*, and 20 min at 10,000 *g*, followed by filtration through a 0.22 μm pore filter. The filtrated medium was ultracentrifuged at 120,000 *g* for 90 min to harvest EVs. The EV fraction was reconstituted with 30 μL of PBS.

### EV isolation from plasma

EV isolation by the ultracentrifugation (UC) method on a sucrose cushion was performed, as previously described [5], with minor modifications. By using ExoQuick solution, EVs were purified by following the manufacturer’s instructions. Briefly, 100 μL of plasma were mixed with ExoQuick solution and incubated at 4°C for 2 h. After centrifugation (1,500 *g*/30 min), the supernatant was discarded and tubes centrifuged again (1,500 *g*/5 min). For the optimized ExoQuick method, after centrifugation (1,500 *g*/30 min), the supernatant was discarded and e-tubes were centrifuged. After precipitation with ExoQuick solution, re-pelleting was performed (1,500 *g*/5 min) three times until the EVs appeared as a beige or white pellet at the bottom of the vessel. These extra steps were needed to reduce contamination with plasma proteins such as albumin. The final pellet was reconstituted with 100 μL of 1x PBS. The schematic comparison of the three protocols is shown in Figure B in S1 File.

### Cell viability

Cell proliferation was assessed by using the 3-[4,5-dimethylthiazol-2-yl]-2,5-diphenyltetrazolium bromide (MTT) reduction assay. Briefly, the proliferation assay was performed by seeding Hep3B, HepG2, and Hepa1-6 cells (1 × 10^4^ cells/well) in a 96-well plate and maintaining them in growth media for 24 h at 37°C under 5% CO_2_. At 80% confluence, the cells were treated with the indicated APAP concentration for 24 h, followed by addition of 20 μL of MTT solution to each well, and then incubated at 37°C for 3 h to allow the production of formazan crystals. Excess MTT solution was removed and filtered DMSO was added to each well to dissolve the formazan crystals. The OD570 nm of each well was read with an ELISA reader (Bio-Rad, Hercules, CA). The results were expressed relative to the control values for each experiment. Cytotoxicity was determined using the MTT reduction method and was also used for calculation of the IC_10, 30, 50_ of APAP.

### Transmission electron microscopy

For negative staining, isolated EVs were fixed in 2.5% glutaraldehyde (vol/vol) in cacodylate buffer. EVs were adsorbed onto 400 mesh carbon-coated copper grids and stained with 0.75% uranyl formate (wt/vol). Samples were observed under a FEI Tecnai G2 Spirit transmission electron microscope (North America NanoPort, Delmont, PA) operated at a 60 kV accelerating voltage. Images were recorded with an Olympus SIS Veleta CCD camera.

### Flow cytometry

For FACS analysis, 10 μg of EVs were incubated with 20 μL of 4-μm diameter aldehyde/sulfate latex beads (Interfacial Dynamics) for 15 min at room temperature in a 30–100 μL final volume, followed by 2 h with gentle shaking in 1 mL PBS. The reaction was stopped by incubation for 30 min in 100 mM glycine. EV-coated beads were washed 3 times in FACS buffer (3% FCS and 0.1% NaN_3_ in PBS) and resuspended in 500 μL FACS buffer. In parallel, EVs were washed twice in FACS buffer. Ten microliters of the coated beads were incubated for 1 h with each primary antibody, followed, when necessary, by incubation in the FITC-conjugated secondary antibody. This was followed by washing and analysis on a FACSCalibur flow cytometer (BD).

### Nanoparticle tracking analysis (NanoSight^™^)

The concentration and diameter of EVs derived from cell culture supernatants or mouse plasma were identified by a NanoSight NS300 system (NanoSight, Amesbury, UK) equipped with a fast video capture and Nanoparticle Tracking analysis (NTA) software[32]. The instrument was calibrated with 100 nm polystyrene beads (Thermo Scientific Fremont, CA) before using. The samples were diluted to 1/1,000 and injected in the 405 nm laser chamber with a constant output controlled by a syringe pump. The samples were captured for 90s at room temperature. NTA software was used to measure the size distribution (in nanometers) and the concentration of nanoparticles (particles/mL). The Batch Process included in the software was used to integrate the three technical measurements of each sample. Each sample was measured three times.

### Immunoblot analysis

Liver tissues, and EV preparations were lysed with RIPA buffer. Protein concentrations were determined using the BCA Protein Assay Kit (Pierce). For immunoblot analyses of plasma-derived EVs, we used the appropriate volumes for 10 μg EV proteins from the control- or APAP-exposed mice. For liver lysates, we analyzed 5 μg proteins for each group. Equal amounts of protein from different samples were separated by SDS/PAGE and transferred to nitrocellulose membranes. They were probed with the respective rabbit polyclonal antibody against CES1 (1:3,000 dilutions; Abcam, Cambridge, MA), APOA1 (1:500 dilutions; Santa Cruz Biotechnology, Santa Cruz, CA), ADH1 (1:3,000 dilutions; Abcam), GST (1:3,000 dilutions; Abcam), ALB (1:1,000 dilutions; Abcam), HP (1:1,000 dilutions; Abcam), FGB (1:5,000 dilutions; Santa Cruz Biotechnology), ERP57 (1:3,000 dilutions; Abcam), PRX1 (1:1,000 dilutions; Abcam), STN1 (1:1,000 dilutions; Abcam), MYL3 (1:3,000 dilutions; Abcam), and FABP3 (1:3,000 dilutions; Abcam). Respective mouse monoclonal antibody against ASS1 (1:2,500 dilutions; Abcam), C3 (1:5,000 dilutions; Santa Cruz Biotechnology), GS (1:1,000 dilutions; Abcam), HSP90 (1:1,000 dilutions; Abcam), β-actin (1:5,000 dilutions; Sigma-Aldrich), HSP60 (1:1,000 dilutions; Abcam), STN1 (1:3,000 dilutions; Abcam), CD63 (1:1,000 dilutions; Abcam), and CD9 (1:3,000 dilutions; Abcam) was also used to detect the specific antigen target, as indicated. Horseradish peroxidase (HRP)-conjugated donkey anti-goat or anti-mouse IgG (Cell Signaling, Danvers, MA, USA) was used as the secondary antibody at a 1:5,000 dilution. Relative protein images were determined by using horseradish peroxidase (HRP)-conjugated secondary antibodies (Amersham International) and ECL substrates (Thermo Fishers). The intensities of the immunoreactive bands were quantified by densitometry using ImageJ software (National Institutes of Health).

### Proteomic analysis by 2D-LC-MS/MS

For proteomic analysis, circulating EVs were purified from the plasma of the APAP-treated mice or control group. The digestion method was processed according to a previously published gel-assisted protocol[30]. Briefly, the total EV pellet was dissolved in a denaturation solution. The proteins were chemically reduced by DTT and alkylated by iodoacetamide (IAA), and the protein solution was mixed with acrylamide/bisacrylamide solution. The gel was cut into small pieces and proteolytic digestion was performed with proteomics-grade trypsin (Qiagen). The tryptic peptides were extracted from the gel using sequential extractions. For the 2D-LC method, nanoscale LC separations of the tryptic peptide mixtures (5 μg of EVs) were performed using the nanoAcquity system (Waters Corporation, Milford, MA) using a hybrid silica XTerra MS C18 column (100 mm × 300 μm, 5 mm) as the first dimensional column and using a Symmetry C18 precolumn (5 μm, 5 mm × 300 μm) and a BEH C18 analytical reversed phase column (1.7 μm, 25 cm × 75 μm) (Waters Corporation) as the second dimensional column, as previously reported[33]. The samples were initially transferred, with an aqueous 0.1% formic acid solution, to the first dimensional column at a flow rate of 0.5 μL/min for 5 min. Mobile phase A consisted of 20 mM ammonium formate and mobile phase B consisted of 20 mM ammonium formate/ACN, pH 10.0, for the first dimensional column. For protein identification, raw data files from MS/MS data were converted into peak lists using Mascot Distiller (Matrix Science; version 2.3.2) using the default parameters. For protein quantification, we used IDEAL-Q software (version 1.0.1.1) to analyze the LC-MS/MS data.

### RNA extraction and real-time analysis

Total RNA was extracted from each mouse liver using Trizol and an RNeasy Mini kit (Qiagen), by following the manufacturer’s instructions. The RNA concentration was measured using a NanoDrop ND-1000 (NanoDrop Technologies, Thermo), and RNA content and quality were analyzed using an Agilent 2100 Bioanalyzer (Agilent Technologies). For real-time analysis, cDNA was transcribed from a total of 600 ng of DNase I-treated RNA by using the cDNA reverse-transcription kit and random primers (Invitrogen). Real-time quantitative reverse-transcriptase polymerase chain reaction (qRT-PCR) was performed using an ABI7500HT system.

### ELISA analysis

The ELISA microplate provided in the commercial kit was pre-coated with an antibody specific to ALB (Abcam) and STN1 (Uscn Life Science, Houston, TX). The plates were blocked for 1 h at 37°C with 200 μL of blocking solution and washed 3 times with PBS with 1% Tween 20 (PBS-T) buffer. EVs and plasma were diluted with PBS-T buffer (90 μL). Ten μLs of the sample were added to the plates in triplicate and incubated for 2 h at room temperature. The plates were reacted with peroxidase-conjugated ALB or STN1 antibody in PBS-T for 30 min and developed with *p*-nitrophenylphosphate. The reaction was stopped and the optical density was measured at 450 nm on an automated iMark instrument (BioRad).

### Statistical analysis

Data are presented as mean ± SD as indicated for each point or graph. Means and standard deviations were calculated using SPSS 17.0 (IBM Inc.). Student’s t-test was used for evaluation of differences in means for normally distributed data. All P values are two-tailed, and values of less than 0.05 were considered to indicate statistical significance. *Significant differences are shown in relation to time- and dose-dependencies at *p* < 0.05 using Tukey’s *post hoc* test and one way-ANOVA (mean ± SD). Other methods not described in this report were as same as the previously described[30, 33, 34].

## Results

### APAP increased EV secretion in rat primary hepatocytes and human hepatocyte cell lines in a dose- and time-dependent manner

**Fig 1:**
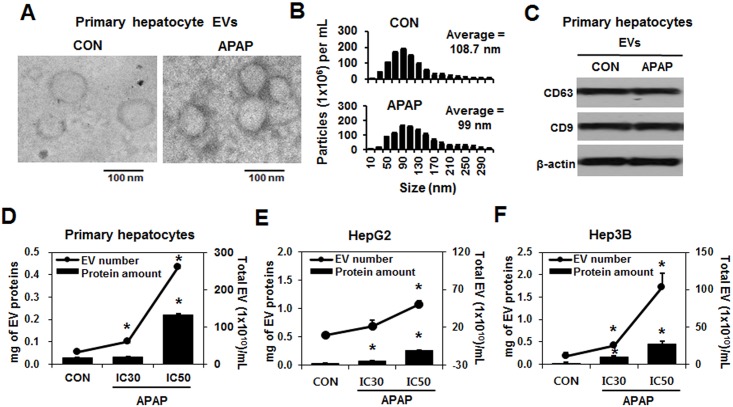
The number and protein content in purified EVs from cultured cells were increased by APAP. Rat primary hepatocytes were exposed to APAP (20 mM APAP) for 24 h (n = 4/group). (A) A representative electron microscopic image of EVs isolated from culture media after primary hepatocytes were exposed to saline (CON) or APAP. (B) Analysis of the size distribution of CON- and APAP-derived EVs. (C) Representative immunoblot analysis of exosome marker proteins in EVs isolated from culture media. (D) Rat primary hepatocytes were exposed to saline (CON), APAP at the IC_30_ (10 mM APAP), or APAP at the IC_50_ (20 mM APAP) for 24 h, and the EVs isolated from the conditioned media (n = 10/group). The numbers and protein amounts in isolated EVs were determined by NanoSight analysis and protein quantification, respectively. (E and F) HepG2 and Hep3B cells were treated with APAP (at the IC_30_ or IC_50_) for 24 h. EVs were isolated from the conditioned media and analyzed using NanoSight analysis and protein quantification, respectively. *P < 0.05.

To evaluate the amounts of EV secreted from hepatocytes by APAP, we treated the rat primary culture hepatocytes with various concentrations of APAP, isolated the EVs from the culture media[31], and confirmed their size and purity by morphological assessment using transmission electron microscopy (TEM). The isolated EVs showed typical rounded morphology (Fig 1A). Nanoparticle tracking analysis of the isolated EVs showed a size distribution consistent with typical exosome vesicles (Fig 1B). Immunoblot analysis showed that the EVs isolated from APAP-treated cells or saline control (CON) contained the well-established exosomal marker proteins such as CD9 and CD63 (Fig 1C). The total number of EV particles per milliliter of culture media of the primary hepatocytes significantly increased by APAP treated for 24 h in a dose-dependent manner (Fig 1D). Similarly, the protein amount in the EVs secreted from rat primary hepatocytes was significantly increased in comparison with the control group in a dose-dependent manner (Fig 1D). The total number of EV particles and the protein amounts in the EVs from HepG2 (Fig 1E and Figure A in S1 File) and Hep3B cells (Fig 1F and Figure A in S1 File) also elevated in a dose- and time-dependent manner following APAP treatment, compared to those of the corresponding control counterparts.

### Optimization of EV isolation method from plasma

Although the ultra-centrifugation (UC)-based method is a gold standard for isolation of EVs, including exosomes from cell culture media, it has been reported that this method may be inappropriate for exosome isolation from plasma because of albumin contamination[35]. Hence, we compared three isolation methods, as illustrated (Figure B in S1 File): the UC-based method[4], use of ExoQuick precipitation solution (EQ)[7], and a new EQ precipitation method (Optimized EQ). The size distribution of EV nanoparticles ranged from 90 to 130 nm, as assessed by NanoSight analysis and TEM (Figure C in S1 File). The expressed levels of CD63 in EVs isolated by the three methods were similar to each other (Figure C in S1 File). However, EVs isolated by EQ and UC were more contaminated with albumin than those isolated by Optimized EQ (Figure C in S1 File). However, the total protein amounts in EVs isolated by Optimized EQ were approximately one half of those purified by the EQ method but was 2-fold greater than those prepared by the UC method (Figure D in S1 File). Based on the purity (Figure C in S1 File and number (Figure D in S1 File) of the EVs, optimized EQ was the most efficient method among the three methods and was therefore chosen for our characterization of EVs isolated from plasma of mouse models of liver injury and human subjects including the alcoholic hepatitis patients. The EV numbers shown in this study may not exact number of EV, because vesicles measured by NTA can include lipid vesicles and proteins in the plasma[32, 36].

### APAP caused hepatocellular necrosis with concomitantly increased circulating EV proteins in mice

**Fig 2:**
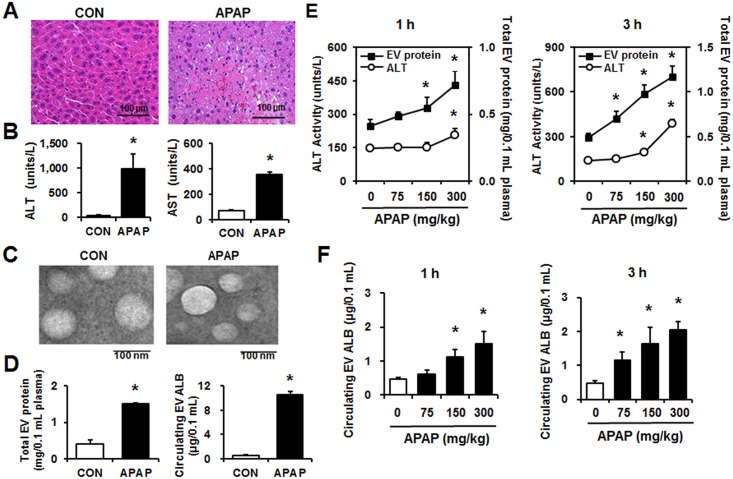
The protein amounts of circulating EVs were increased in APAP-exposed mice. Wild-type male BALB/c mice (6 weeks old) received a single ip injection of APAP (300 mg/kg) or saline control (CON) and were sacrificed after 24 h (n = 10/group). (A) Representative hematoxylin and eosin (H&E) staining of formalin-fixed liver sections. (B) Plasma ALT and AST levels. (C) Representative electron microscopic images of EVs purified from mouse plasma. (D) Analysis of the protein amounts in circulating EVs. The amounts of a liver-specific protein ALB were measured by ELISA specific for mouse ALB. (E) Wild-type male BALB/c mice (6 weeks old) were injected with a single dose of 75, 150, or 300 mg/kg APAP, and the protein amounts in circulating EVs were measured after 1 or 3 h (n = 10/group). The ALT levels are shown on the left while the circulating EV protein amounts are indicated on the right side of each Figure. (F) The amounts of albumin (ALB) in circulating EVs, isolated from plasma of the indicated groups, were measured by ELISA. *P < 0.05.

Histopathologic examination of mice exposed to 300 mg/kg APAP (LD_50_) revealed hepatocellular necrosis in the centrilobular region (Fig 2A). Plasma samples from APAP-treated mice showed significantly elevated ALT and AST levels in comparison with the control samples (Fig 2B). The protein levels of fatty acid synthase (FAS) and cytochrome C (CYTC) moderately elevated while the mRNA levels of tumor necrosis factor-alpha (TNF-α) significantly increased in the livers of APAP-treated mice compared to the saline controls (Figure E in S1 File).

We isolated EVs from the plasma of CON- or APAP-exposed mice by the optimized EQ method and confirmed their morphology by TEM (Fig 2C). Nanoparticle tracking analysis of isolated EVs showed a size distribution consistent with typical exosomes (Figure F in S1 File). In contrast, endoplasmic reticulum markers endoplasmic reticulum protein 57 (ERP57) and calnexin (CANX) were not detectable in circulating EVs, but were detected in whole liver lysates, regardless of APAP treatment (Figure F in S1 File). To rule out contaminants such as apoptotic blebs, we loaded the EVs purified from the plasma samples onto a discontinuous sucrose gradient. Immunoblot analysis demonstrated the presence of exosomal marker CD63 in sucrose density fractions 7 and 8, whereas the apoptotic bleb marker prohibitin was not detected in any fraction with the anti-prohibitin antibody, although this antibody clearly detected prohibitin in the MCF-7 cell lysates, used as a positive control (Figure G in S1 File). APAP exposure (300 mg/kg for 24 h) significantly elevated the amount of circulating EV proteins in comparison with control (CON) mice (Fig 2D, left panel). In addition, ELISA results showed that the amount of a liver-specific protein albumin (ALB) in the EVs markedly increased in the APAP-treated mice (Fig 2D, right panel). In contrast, the amounts of ALB in the plasma were unchanged by APAP exposure (Figure H in S1 File). EV number showed a significant correlation with ALB protein level in circulating EVs (Figure I in S1 File).

To further support the results shown in Fig 2A–2D, different mice were exposed to a single injection with a different dose of APAP (i.e., 75, 150, or 300 mg/kg) for 1 h and 3 h. As expected, pathological changes in the liver occurred in a dose- and time-dependent manner (Figure J in S1 File). ALT levels were elevated in a dose- and time-dependent manner, particularly at 1 and 3 h following treatment with 300 mg/kg APAP (Fig 2E). Changes in EV protein amounts also showed better correlations with APAP dosage and exposure time than with ALT changes (Fig 2E). Furthermore, mouse ALB levels in circulating EVs, quantified by ELISA, were significantly increased by APAP in a dose- and time-dependent manner (Fig 2F). These results with two separate mouse experiments strongly indicate a utility of liver-specific EV proteins, including albumin (ALB), being used as a potential biomarker for DILI.

### Proteomic analysis of circulating EV proteins from control and APAP-exposed mice

**Fig 3:**
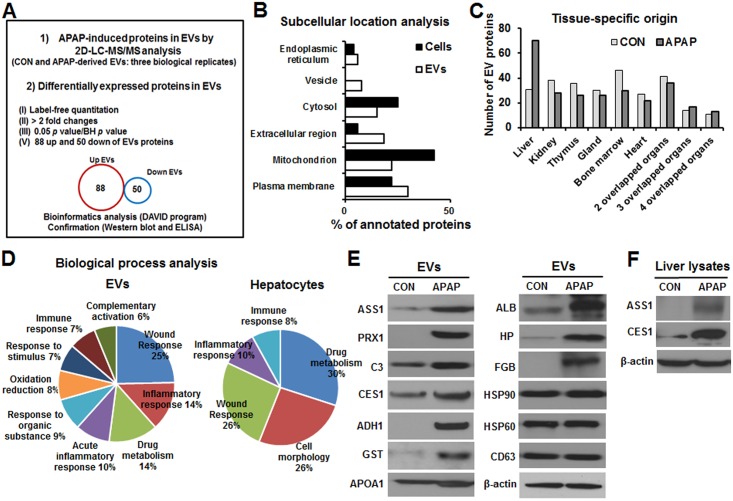
Proteomic analysis of circulating EVs isolated from APAP-exposed mice and confirmation of various proteins by immunoblot analysis. (A) Proteomic analysis of EV proteins isolated from Control or APAP-exposed mice using 2D LC-MS/MS. Differentially expressed proteins were identified by bioinformatic analysis, as described. (B) Classification based on the subcellular location of the identified proteins. Comparison of proteomic data from the EVs in this study with those of rat primary hepatocytes, as reported previously by our group[30]. (C) The 380 proteins in EVs from control plasma and 431 proteins in EVs from APAP plasma were classified by tissuespecific origin and categorized according to the DAVID program. Some proteins are classified to more than two organs. (D) Comparison of differentially expressed proteins in circulating EVs from APAP-treated groups with the previously reported results[39] from hepatocytes treated with APAP, based on biological process analysis. (E) Confirmation of liver-specific proteins, inflammation-related proteins, and exosomal marker proteins in circulating EVs by immunoblot analysis, as indicated. (F) Protein levels of ASS1 and CES1 were measured in whole liver lysates from the indicated groups.

Circulating EVs were purified from the plasma of the APAP-treated mice and control group. EV proteins were then digested with trypsin and analyzed by nano-LC-MS/MS (Fig 3A)[37]. The analysis of 3 biological replicates identified 4,260 unique peptides corresponding to 679 distinct proteins (p < 0.05, protein score ≥ 34, false discovery rate = 0.5%). After stringent filtering criteria were applied (adjusted *p-*value [BH *p-*value] <0.05 and >2-fold expression difference), 138 proteins were determined to be differentially expressed between the two groups (Table A in S1 File). The 99 proteins, identified in the APAP-induced EVs, were determined as EV-specific proteins based on ExoCarta database, while 23 proteins in the APAP-induced EVs were consistently present in EVs from hepatocytes[38] (Table A in S1 File). The liver-specific proteins are alcohol dehydrogenase-1 (ADH1), glutathione-*S* transferase (GST), albumin (ALB), haptoglobin (HP), fibrinogen (FGB), etc.

These data, based on the subcellular locations of the identified proteins, were compared with our previously-reported proteomic data from primary hepatocytes[30]. Vesicular proteins were detected, and the portions corresponding to plasma membrane proteins and extracellular proteins were greater in the EV proteome than in the hepatocellular proteome, which contains mostly cytosolic and mitochondrial proteins (Fig 3B).

We manually classified the identified EV proteins based on organ specificity using information from the DAVID program (http://david.abcc.ncifcrf.gov). The number of liver-specific proteins, such as liver carboxylesterase-1 (CES1), apolipoprotein A-1 (APOA1), ADH1, GST, ALB, HP, and FGB, increased by more than 2 folds in APAP-derived EVs, whereas the numbers of proteins specific to other organs were similar between APAP- and CON-derived EVs (Fig 3C). The differentially expressed proteins (138 proteins) were involved in the wound response, the inflammatory response, drug metabolism, acute inflammatory response, etc (Fig 3D, left panel). Some of the identified pathways, including those related to the wound response, the inflammatory response, drug metabolism, the acute inflammatory response, and oxidation reduction, are commonly identified in hepatocytes treated with APAP (Fig 3D, right panel)[39]. We confirmed the upregulation of inflammatory-related proteins such as argininosuccinate synthase-1 (ASS1), peroxiredoxin-1 (PRX1), and complement component-3 (C3) (Fig 3E), as well as hepatic cytosolic proteins such as CES1, ADH1, and GST, which are involved in xenobiotic, detoxification, and drug metabolism processes (Fig 3E). By immunoblot analyses, we further validated secreted, liver-specific proteins such as APOA1, ALB, HP, and FGB, which are tentative hepatotoxic markers during hepatic damage (Fig 3E). Exosomal marker proteins such as HSP90, HSP70, and CD63 were also validated (Fig 3E). Upregulation of ASS1 and CES1 proteins was also observed in the liver extracts of APAP-exposed mice (Fig 3F).

### Prevention of liver injury decreased increased EV secretion by APAP

**Fig 4:**
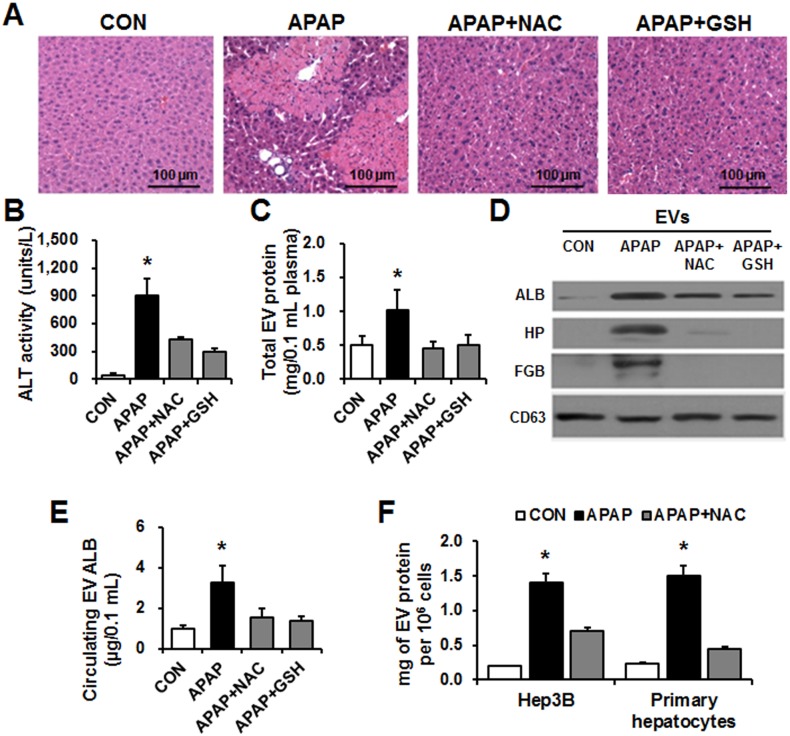
Analysis of total proteins and albumin amounts in circulating EVs after prevention of liver injury by NAC or GSH. Acute liver injury was induced in BALB/c male mice by a single i.p. injection of APAP (300 mg/kg), and some of the mice received 10 mL/kg saline, 100 mg/kg NAC, or 200 mg/kg GSH intravenously 1.5 h after APAP administration (n = 10/group). (A) Representative H&E staining of formalin-fixed liver sections. (B) Plasma ALT levels. (C) Protein amounts in circulating EVs derived from each indicated group were calculated. (D) Detection of liver-specific proteins in circulating EVs by immunoblot analyses, as indicated. (E) The amounts of mouse ALB in EVs from each group were measured by ELISA. (F) Hep3B and primary hepatocyte cells were exposed to APAP (IC_50_) without or with NAC (5 mM) for 24 h (n = 4/group). The amounts of EV proteins isolated from Hep3B and primary hepatocyte cells treated with APAP alone or APAP+NAC were measured. *P < 0.05.

To substantiate the finding that increased liver-specific EV proteins are specifically caused by liver injury, we measured the protein levels in circulating EVs in APAP-exposed mice with or without liver injury by treatment with anti-oxidant *N*-acetylcysteine (NAC) or glutathione (GSH), which can prevent APAP-induced hepatotoxicity[13]. Mice were treated with NAC or GSH via intravenous administration at 1.5 h after APAP treatment and allowed to live for additional 24 h[40]. NAC- or GSH-treated mice showed markedly fewer numbers of necrotic hepatocytes and inflammatory cells in comparison with those treated APAP alone (Fig 4A). Hepatoprotection was also observed in APAP-exposed mice when they were treated with NAC or GSH, as demonstrated by the significant decrease in serum ALT levels at 6 h (Fig 4B). Our results showed that treatment with NAC or GSH significantly reduced the protein contents in circulating EVs nearly similar to the basal levels of the control group (Fig 4C). The levels of 3 representative liver-specific proteins (i.e., ALB, HP, and FGB) in the EVs from the indicated groups were analyzed by immunoblot analyses. APAP-induced increases in ALB, HP, and FGB levels in circulating EVs were significantly abolished or reduced to saline-treated control levels in the NAC- and GSH-treated groups (Fig 4D), suggesting that the increased EV proteins in the APAP-treated mice correlated with the severity of liver-specific injury. In addition, ELISA for mouse ALB revealed significantly decreased levels of EV ALB in the NAC- or GSH-treated groups, compared to those of mice treated with APAP alone (Fig 4E). To further demonstrate the reversible changes of EV proteins in an *in vitro* model, Hep3B cells and primary hepatocytes were treated with APAP in the absence or presence of NAC co-treatment. NAC-treated cells showed significantly lower levels of EV proteins than those isolated from cultured HepG3 cells and primary hepatocytes exposed to APAP alone (Fig 4F).

### Myotoxic bupivacaine (BPVC) elevated muscle-specific proteins in circulating EVs, but not liver-specific proteins

**Fig 5:**
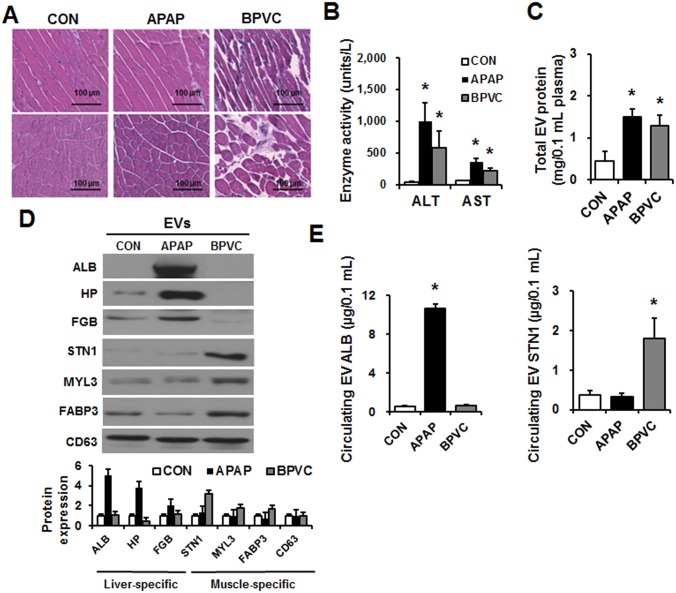
Analysis of liver- or muscle-specific proteins in circulating EVs from mice with liver or muscle injury. Wild-type male BALB/c mice (6 weeks old) were treated with a single ip injection of APAP (300 mg/kg) or intramuscular injection with 0% or 0.5% BPVC into the right and left tibialis anterior muscles and were sacrificed at 24 h post treatment (n = 10/group). (A) Representative H&E staining of formalin-fixed muscle sections for all indicated groups. (B) Plasma ALT and AST levels. (C) Analysis of the total protein amounts in circulating EVs isolated from the indicated groups. (D) Immunoblot analyses of liver-specific or muscle-specific proteins in circulating EVs from each group, as indicated. (E) The amounts of ALB or STN1 in circulating EVs from each group were respectively measured by ELISA specific for mouse proteins. *P < 0.05.

Elevated plasma ALT levels after liver injury and muscle injury have been well-established[15]. To correlate the specificity of EV proteins with increased ALT in skeletal muscle injury, bupivacaine-HCl (BPVC) was used to stimulate skeletal muscle injury in mice[6]. We determined the levels of serum enzymes (i.e., ALT and AST) and quantified the amounts of proteins in circulating EVs. Histopathological analysis revealed clear evidence of myotoxicity (Fig 5A) by BPVC, which did not cause hepatotoxicity (Figure K in S1 File). BPVC treatment induced a modest, but statistically significant elevation in serum ALT and AST levels compared to controls (Fig 5B), as previously reported[6]. The total EV protein contents were significantly increased in both APAP-induced liver injury and BPVC-induced muscle injury (Fig 5C). However, EVs from APAP-treated mice showed increased levels of liver-specific proteins, such as ALB, HP, and FGB, but not the muscle-specific proteins (Fig 5D). These liver-specific proteins in circulating EVs were not detected in BPVC-exposed mice with myotoxicity. On the other hand, circulating EVs isolated from BPVC-treated mice showed increased levels of the muscle-specific proteins, but not of the liver-specific proteins (Fig 5D). Skeletal muscle troponin-1 (STN1), myosin light chain-3 (MYL3), and fatty acid binding protein-3 (FABP3) are widely accepted biomarkers for skeletal muscle injury[41]. Furthermore, ELISA results showed that liver-specific EV proteins, including mouse ALB, were observed in APAP-exposed mice while muscle-specific protein STN1 was detected in circulating EVs from BPVC-exposed mice, but not in EVs prepared from APAP-exposed mice (Fig 5E). These results indicate a highly specific pattern of elevated EV proteins in a tissue-dependent manner, further supporting the utility of liver-specific EV proteins as biomarkers of liver injury.

### Liver-specific EV proteins were increased by other hepatotoxic agents thioacetamide and d-galactosamine, but not by nonhepatotoxic penicillin

**Fig 6:**
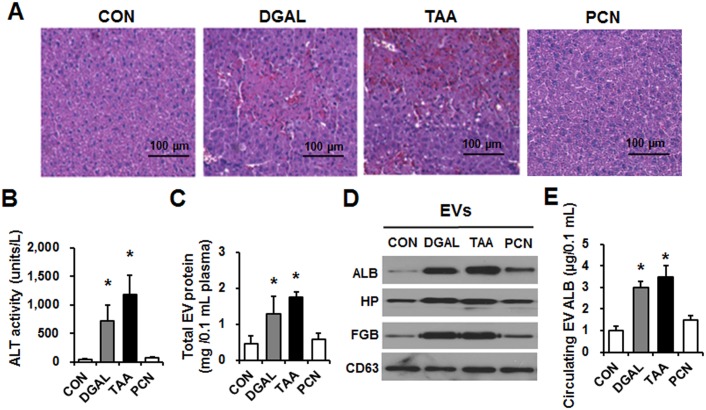
Analysis of total protein amounts in circulating EVs from DGAL-, and TAA- treated mice. Wild-type male BALB/c mice (6 weeks old) were treated a single ip injection with PBS, DGAL (1,000 mg/kg), TAA (200 mg/kg), or PCN (2,400 mg/kg, control group) for 24 h (n = 10/group). (A) Representative H&E staining of formalin-fixed liver sections. (B) Plasma ALT level. (C) Protein amounts in circulating EVs were measured. (D) Detection of liver-specific proteins in circulating EVs by immunoblot analyses for each target protein. (E) The amounts of liver-specific mouse ALB in EVs from the indicated groups were measured by ELISA. *P < 0.05.

To further demonstrate the tissue specificity of EV proteins, mice were treated with thioacetamide (TAA) or d-galactosamine (DGAL), which can induce acute liver injury and cause significant DILI[42]. Penicillin (PCN), a widely-used antibiotic, was chosen as a negative control agent[43]. We confirmed that histopathological analysis of mouse livers verified hepatic injury caused by TAA or DGAL, but not by PCN (Fig 6A). Serum ALT levels were significantly elevated in TAA- and DGAL-exposed mice, but not after PCN treatment (Fig 6B). Circulating EVs were isolated from the plasma of mice treated with each indicated drug. The total protein contents in circulating EVs significantly elevated by 2.9- and 3.8-fold in mice treated with DGAL and TAA, respectively, but not in mice treated with PCN (Fig 6C). In addition, the levels of the 3 representative liver-specific proteins were significantly elevated in the DGAL- or TAA-treated group but not in the PCN-treated mice (Fig 6D). Furthermore, the amounts of ALB in the circulating EVs, as measured by ELISA for mouse ALB, were significantly increased in the TAA- or DGAL-treated mice but not in the PCN-exposed group (Fig 6E).

### Ethanol elevates liver-specific EV proteins in mice and human individuals

**Fig 7:**
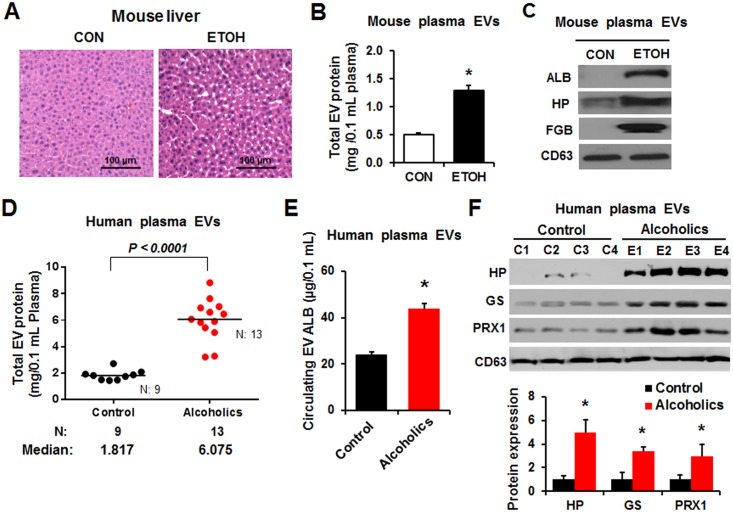
The amounts of total and liver-specific proteins in circulating EVs were increased in binge ethanol-exposed mice and alcoholics. (A) Wild-type male BALB/c mice (6 weeks old) were treated twice with saline (CON) or 6 g ethanol/kg via oral gavage at 12-h interval and were sacrificed 1 h after the second dose (n = 10/group). Representative H&E staining of formalin-fixed liver sections. (B) Protein amounts of the circulating EVs isolated from control or alcohol-exposed mice, as indicated. *P < 0.05. (C) Detection of the liver-specific proteins in circulating EVs from the indicated mouse plasma by immunoblot analysis. (D) Analysis of the protein amounts in circulating EVs isolated from the sera of healthy controls (n = 9), who never drank alcohol due to religious reasons, and alcoholics (n = 13) with hepatitis. (E) The amounts of liver-specific protein ALB in circulating EVs from the indicated groups were measured by ELISA for human ALB protein. *P < 0.05. (F) Detection of the liver-specific proteins in circulating EVs from the different groups by immunoblot analyses, as indicated. *P < 0.05.

To further demonstrate the utility of liver-specific EV proteins as potential biomarkers of liver injury, we also evaluated additional models of liver injury in alcohol-exposed mice and alcoholic people with hepatitis. Male mice were exposed to two doses of ethanol (6 g/kg/each via oral gavage) at 12-h interval and then sacrifice at 1 h after the last dose of ethanol. Binge alcohol-exposed mice showed slightly or moderately increased fat accumulation in the liver (Fig 7A), as recently reported[17]. The amounts of the total (Fig 7B) and liver-specific proteins such as ALB, HP and FGB (Fig 7C) in circulating EVs from binge alcohol-fed mice were significantly elevated compared to those of dextrose-fed control mice (Fig 7B and 7C, respectively). Similar results of increased total EV proteins have been observed in binge alcohol-fed Fishers rats, in comparison to those of dextrose-fed control rats (data not shown).

To verify whether the elevated total and liver-specific proteins observed in alcohol exposed animals can be replicated with human samples, we isolated circulating EVs, from sera from healthy control people and alcoholics who suffered alcoholic hepatitis (Table 1), and subsequently studied their properties. The total EV protein contents were significantly higher in the sera from alcoholics than the controls (Fig 7D). In addition, ELISA specific for human ALB showed significantly elevated amounts of a liver-specific protein ALB in the EVs of alcoholics compared with those of control people (Fig 7E). In addition, immunoblot analyses revealed that significantly increased levels of liver-specific proteins, such as HP (haptoglobin), GS (glutathione synthetase), and PRX1 (peroxiredoxin 1), were observed in the circulating EVs from alcoholics compared to those of controls (Fig 7F). These results indicate that increased amounts of total and liver-specific proteins in circulating EVs were consistently observed in various animal models and human specimens in a species-independent manner and that the liver-specific exosomal proteins could be used as potential non-invasive biomarkers for drug- or alcohol-induced liver injury.

## Discussion

Over the past decade, omics technologies have shown promising results for improving current liver toxicity tests[44]. Researchers have developed microarrays to profile gene expression changes and mass spectrometry to quantify differential protein expression after xenobiotic exposure, often referred to as toxicogenomics and toxicoproteomics, respectively. These powerful tools have been increasingly used to investigate the molecular mechanistic processes underlying drug-induced organ injury[44, 45]. Our laboratory has also reported the mechanism of hepatotoxicity cause by a high dose of cisplatin[30], simvastatin[33], and azathioprine[34] by using integrated transcriptomic and proteomic approaches. We provided the first pathway map, related to cisplatin-induced liver injury, offering new insights into the hepatotoxicity mechanism with the integrated proteomic and genomic approaches[30]. In addition, miR-122[27], high mobility group box-1 (HMGB1)[27], keratin-18[27], mitochondrial DNA (mtDNA)[46], glutamate dehydrogenase (GLDH)[27], and carbamoyl phosphate synthetase-1 (CPS1)[47] have recently been proposed as sensitive biomarkers of hepatotoxicity in clinical reports. However, additional research is needed before they are accepted in clinical and regulatory settings.

EVs from readily-available biofluids, such as blood and urine, have been reported as the sources of molecular biomarkers for the early detection and prognosis of various diseases[4, 8]. This approach is based on the observation that EVs present in biofluids contain tissue-specific proteins and miRNAs derived from certain organs, and that the exosome number and contents may be fluctuated depending on a given disease state[4, 8]. Currently, serum ALT/AST levels have been widely used as clinical markers of liver injury; although the ALT/AST measurements require fresh blood samples and lack tissue specificity, since elevated serum ALT and AST levels could be also observed in experimental models and patients with muscle or kidney diseases. In addition, liver disease without elevated ALT/AST levels can be observed in some cases, as reported[48, 49]. Thus, due to the lack of tissue or disease specificity, there is a need for more sensitive, stable, and specific biomarkers of liver injury[15]. For instance, serum ALT has a half-life of about 13 h in circulation[15], and the newly proposed liver injury biomarker miR-122 has an even shorter half-life of several hours[24, 50]. Therefore, false negatives can be potentially observed when a critical time window of injury detection is missed by using short-lived biomarkers such as serum ALT and miR-122. In contrast, circulating EV-based biomarkers for drug- or alcohol-induced liver injury can be better alternatives due to longer half-lives of EV proteins, since they are protected from proteolytic degradation[8].

Hepatocytes constantly produce EVs that are released into circulation to transport signaling molecules and cellular waste[51]. Based on readily availability and relative stability, circulating EVs are being vigorously explored as biomarkers of many disease states and toxic conditions, including drug- or alcohol-induced liver injury[8]. Emerging data suggest that blood- and urine-derived EVs contain mRNA and miRNA, while their levels can be remarkably elevated in response to liver injury. Furthermore the increased amounts of circulating EVs correlate well with classical measures of liver damage[8]. In the DGAL model and in APAP-treated rats, the levels of EV-associated liver-specific mRNA transcripts (e.g., ALB, HP, and FGB) in the serum increased and were correlated with evidence of liver injury[6]. Using a DGAL-induced acute liver failure model, it was found that serum-derived EVs from treated rats contained significantly increased levels of albumin mRNA[52]. In addition, the amount of liver-specific miRNA-122 was elevated in plasma and circulating EVs after drug- or alcohol-induced liver injury[7]. In animal models of liver injury, changes in the number and content of liver-derived EVs were also observed in urine[53].

In this work, we showed that the amounts of total and liver-specific proteins in circulating EVs, characterized by three different purification methods and TEM, were significantly elevated in acute liver injury caused by APAP or binge alcohol. Elevated amounts of EV proteins can be also detected in APAP-exposed cultured cells of hepatic origin. Similar results were consistently observed in experimental rodents (e.g., mice and rats) and alcoholic people with hepatitis compared to those of their respective controls. In addition, treatment with an antioxidant NAC or GSH not only blocked the APAP-mediated liver injury but also prevented elevation of liver-specific proteins in EVs, suggesting that increased oxidative stress is likely to play a role in promoting liver injury and secretion of hepatic proteins into circulating exosomes. Our preliminary results and ongoing study obtained with cultured hepatocytes also showed that oxidative hepatic injury is required for increased amounts of exosomal proteins. Although the detailed mechanism of increased EVs in liver injury needs further investigation, non-the-less, our mass-spectrum based proteomic analysis further demonstrated that circulating EVs from APAP-treated mice predominantly contained liver-specific proteins. In our study, APAP treatment affected the abundance of 138 proteins in circulating EVs. Some of these EV proteins include the proteins associated with membrane structure, inflammation, drug and intermediary metabolism, and vesicle formation. Remarkably, liver-specific proteins such as CES1, ADH1, GST, APOA1, ALB, HP, and FGB in the EVs increased after APAP-induced liver injury. Similar results of elevated liver-specific EV proteins were also observed in acute liver injury caused by DGAL or TAA but neither by non-hepatotoxic drug penicillin nor myotoxicity induced by BPVC, which selectively elevated muscle-specific proteins in circulating EVs, without increasing liver-specific proteins. These results strongly support the utility of EV proteins as specific biomarkers of acute liver injury or muscle injury, depending on the property of a drug or agent. These data also show a pattern similar to that reported by the Falcon-Perez group[54], who reported that larger numbers of hepatic enzymes were found in rat primary hepatocyte-derived EVs after DGAL- and LPS-induced liver injury, and that these enzymes were involved in endogenous and xenobiotic metabolism. Here, we also showed that liver-specific proteins such as ALB, HP, and FGB in the circulating EVs increased in the alcohol-induced liver injury of rodent models. The results in experimental cell and animal models were further verified with the EVs isolated from control people and alcoholics. To our knowledge, our study is the first report to demonstrate the elevation of total and some liver-specific proteins in circuiting EVs after alcohol feeding or ingestion. These methods are based on the increased amounts of liver-specific EV proteins, and thus overcome the limitations of assessment using ALT[15], which can show non-specific elevation, due to its increment after muscle injury, or absence of its elevation, due to relatively short half-life of ALT. Furthermore, EV proteins have much greater stability with longer half-lives due to protection from proteolytic degradation^1^. Based on these facts, our results suggest that the assessment of a few liver-specific EV proteins as noninvasive biomarkers of liver injury is promising.

Regarding the functional implications of EVs secreted from each organ, recent reports suggest that intercellular communication by EVs can trigger various signal-transduction pathways that modulate cellular response or behavior [55]. The role of these signaling events may be to coordinate adaptive cellular responses that maintain tissue homeostasis[55]. Our study demonstrates that liver-specific proteins in circulating EVs can be used as potential biomarkers to detect liver damage caused by hepatotoxic drug- or alcohol exposure. Circulating EVs secreted from liver injury may play a role in cell-cell communications. Although the role of oxidative stress and the mechanisms of elevated EV secretion and protein contents in alcohol-induced animal models are being investigated in our laboratory, to our knowledge, this study represents the first report to show that APAP- and ethanol-induced liver injury increase the amounts of total and liver-specific proteins in EVs, further supporting the utility of detecting EV proteins as reliable and noninvasive biomarkers of liver injury. Furthermore, this conclusion was validated by the analysis results of serum specimens from human alcoholics and control individuals.

## Supporting information

S1 File **Table A in S1 File. List of differentially expressed proteins in APAP-derived EVs based on biological function. Figure A in S1 file. The number and protein amount of EVs prepared from liver cell lines are increased by APAP.** HepG2 and Hep3B cells were treated with CON (growth media) or APAP at the IC_30_ dose, or APAP at the IC_50_ dose for 1, 3, 12, or 24 h, as indicated, and the EVs isolated from the culture supernatants (n = 3/group). (A) The numbers of EVs isolated from the HepG2 cell culture media were determined by NanoSight analysis. (B) The protein amounts in EVs, isolated from the HepG2 cell culture media, were quantified using protein analysis. (C) The numbers of EVs isolated from the Hep3B cell culture media were determined by NanoSight analysis. (D) The protein amounts in EVs, isolated from the Hep3B cell culture media, were quantified using protein analysis. **Figure B in S1 File. Schematic overview of the 3 methods of EV isolation from mouse plasma. Figure C in S1 File. Characteristics of plasma-derived EVs isolated by the three different methods.** EVs were isolated from plasma of control mice by the three different methods, as indicated. (A, B) The size profiles of EVs have been evaluated by NanoSight analysis (A) and TEM imaging (B). (C) Immunoblot analyses were performed with 20 μg proteins/well to determine the relative levels of CD63, ERP57, and albumin in plasma-derived EVs prepared by the three different methods. **Figure D in S1 File. Comparison of the number and total proteins in EVs isolated by ExoQuick, Optimized ExoQuick, and UC methods.** EVs were isolated from plasma of control mice by the indicated methods. (A) The protein amounts in EVs were isolated from the plasma by ExoqQuick, Optimized ExoQuick, and UC methods are presented (n = 3/group). (B) The numbers of EVs isolated from the plasma using the three methods were determined by NanoSight analysis (n = 3/group). **Figure E in S1 File. APAP exposure induced liver injury in mice.** Wild-type male Balb/C mice (6 weeks old) were injected with a single ip dose of APAP (300 mg/kg, LD_50_ dosage) or saline (CON, negative control) for 24 h to produce APAP-induced hepatic injury. (A, B) The protein levels of cytochrome C (CYTC) and fatty acid synthase (FAS) (A) and mRNA level of TNF-α (B) in whole liver lysates were measured (n = 4/group), as indicated. Data represent the mean ± SD. *P < 0.05. **Figure F in S1 File. Characteristics of circulating EVs from mouse plasma.** Wild-type male Balb/C mice (6 weeks old) were injected with a single ip dose of saline (CON) or APAP at 300 mg/kg for 24 h. (A) The number and size (nm) distribution of EVs isolated from plasma in CON and APAP-treated mice were determined by Nanoparticle Tracking Analysis (NanoSight). (B) Representative western blot analyses for the indicated proteins in circulating EVs and whole liver lysates. Endoplasmic markers, endoplasmic reticulum protein 57 (ERP57) and calnexin (CANX), were not detected in circulating EVs, but detected in whole liver lysates. **Figure G in S1 File. Confirmation of EV marker protein in circulating EVs purified from mouse plasma on a discontinuous sucrose gradient.** (A, B) Plasma-derived circulating EVs from control mice were loaded on a discontinuous sucrose gradient (0.94–1.25 g/mL). Eleven fractions were collected. Immunoblot analysis were performed to verify the distribution of EV proteins on the sucrose gradient by detecting CD63 as a marker of EV protein. Immunoblot analysis revealed that prohibitin, an apoptosis bleb marker, was not associated with EV fractions, although it was detected in MCF cell lysates, used as a positive source. **Figure H in S1 File. The amount of mouse plasma ALB was not changed by APAP. Figure I in S1 File. The correlation of EV protein with EV ALB level was identified by correlation test. Figure J in S1 File. APAP induces centrilobular hepatocellular necrosis in a dose- and time-dependent manner.** Male BALB/c mice (6 weeks old) were treated with a single ip injection of 75, 150, or 300 mg/kg APAP for 1 or 3 h (n = 10/group). Representative H&E stained slides of formalin-fixed liver sections of the indicated groups are presented. Scale bars, 100 μm. **Figure K in S1 File. Myotoxic BPVC does not induce centrilobular hepatocellular necrosis.** Male BALB/c mice (6 weeks old) were treated with 0 (CON) or 0.5% BPVC via intramuscular injection and sacrificed after 24 h. Representative H&E staining of formalin-fixed liver slides of the indicated groups are shown (n = 10/group). Scale bars, 100 μm.
(DOCX)

## Abbreviations

ADH1: alcohol dehydrogenase-1
ALB: albumin
ALD: Alcoholic liver disease
ALT: alanine aminotransferase
APAP: acetaminophen
APOA1: apolipoprotein A-1
ASS1: argininosuccinate synthase-1
AST: aspartate aminotransferase
BPVC: bupivacaine-HCl
C3: complement component-3
CES1: liver carboxylesterase-1
DGAL: d-galactosamine
DILI: drug-induced liver injury
EVs: extracellular vesicles
FABP3: fatty acid binding protein-3
FGB: fibrinogen
GS: glutathione synthetase
GSH: glutathione
GST: glutathione S-transferase
HP: haptoglobin
MYL3: myosin light chain-3
NAC: *N*-acetylcysteine
PRX1: peroxiredoxin-1
STN1: skeletal muscle troponin-1
TAA: thioacetamide
TEM: transmission electron microscopy
