# Supplementary material for: Increased liver-specific proteins in circulating extracellular vesicles as potential biomarkers for drug- and alcohol-induced liver injury
Source: PLoS One. 2017 Feb 22;12(2):e0172463. doi: 10.1371/journal.pone.0172463 (PMC5321292; doi:10.1371/journal.pone.0172463)
Supplement: S1 File — Table A in S1 File. List of differentially expressed proteins in APAP-derived EVs based on biological function. Figure A in S1 file. The number and protein amount of EVs prepared from liver cell lines are increased by APAP. HepG2 and Hep3B cells were treated with CON (growth media) or APAP at the IC30 dose, or APAP at the IC50 dose for 1, 3, 12, or 24 h, as indicated, and the EVs isolated from the culture supernatants (n = 3/group). (A) The numbers of EVs isolated from the HepG2 cell culture media were determined by NanoSight analysis. (B) The protein amounts in EVs, isolated from the HepG2 cell culture media, were quantified using protein analysis. (C) The numbers of EVs isolated from the Hep3B cell culture media were determined by NanoSight analysis. (D) The protein amounts in EVs, isolated from the Hep3B cell culture media, were quantified using protein analysis. Figure B in S1 File. Schematic overview of the 3 methods of EV isolation from mouse plasma. Figure C in S1 File. Characteristics of plasma-derived EVs isolated by the three different methods. EVs were isolated from plasma of control mice by the three different methods, as indicated. (A, B) The size profiles of EVs have been evaluated by NanoSight analysis (A) and TEM imaging (B). (C) Immunoblot analyses were performed with 20 μg proteins/well to determine the relative levels of CD63, ERP57, and albumin in plasma-derived EVs prepared by the three different methods. Figure D in S1 File. Comparison of the number and total proteins in EVs isolated by ExoQuick, Optimized ExoQuick, and UC methods. EVs were isolated from plasma of control mice by the indicated methods. (A) The protein amounts in EVs were isolated from the plasma by ExoqQuick, Optimized ExoQuick, and UC methods are presented (n = 3/group). (B) The numbers of EVs isolated from the plasma using the three methods were determined by NanoSight analysis (n = 3/group). Figure E in S1 File. APAP exposure induced liver injury in mice. Wild-type male Balb [file pone.0172463.s001.docx]

**Table A in S1 File. List of differentially expressed proteins in APAP-derived EVs based on biological function.**

| **IPI NO.** | **Gene name** | **Protein description** | **Ratio^a^ (APAP/CON)** | **SD** | **Exo**  **Carta^b^** | **Presence in EVs from hepatocytes^c^** | **Presence in EVs from HepG2 cells^d^** |
| --- | --- | --- | --- | --- | --- | --- | --- |
|  |  | **Apoptosis** |  |  |  |  |  |
| IPI00229727 | Bag5 | BAG family molecular chaperone regulator 5 | 2.2 | 0.3 | **O** |  |  |
| IPI00117264 | Park7 | Protein DJ-1 | 0.1 | 0.1 | **O** |  |  |
| IPI00130039 | Pskh1 | Serine/threonine-protein kinase H1 | 0.4 | 0.2 |  |  |  |
|  |  | **Carbohydrate and amino-acid metabolism** | |  |  |  |  |
| IPI00153317 | Aldh1L1 | 10-formyltetrahydrofolate dehydrogenase | 2.0 | 0.1 | **O** | **O** | **O** |
| IPI00134746 | Ass1 | Argininosuccinate synthase | 4.3 | 0.4 | **O** | **O** | **O** |
| IPI00318841 | Eef1g | Elongation factor 1-gamma | 0.5 | 0.1 | **O** | **O** | **O** |
| IPI00454049 | Echs1 | Enoyl-CoA hydratase, mitochondrial | 2.0 | 0.3 | **O** |  | **O** |
| IPI00129276 | Eif3a | Eukaryotic translation initiation factor 3 subunit A | 0.2 | 0.2 | **O** |  |  |
| IPI00856379 | Aldoa | Fructose-bisphosphate aldolase | 0.2 | 0.1 | **O** | **O** | **O** |
| IPI00319994 | Ldha | L-lactate dehydrogenase A chain | 6.0 | 0.3 | **O** | **O** |  |
|  |  | **Cytoskeleton-related protein** |  |  |  |  |  |
| IPI00115627 | Actr3 | Actin-related protein 3 | 0.2 | 0.2 | **O** |  |  |
| IPI00266188 | Cfl2 | Cofilin-2 | 4.0 | 0.4 | **O** |  |  |
| IPI00119876 | Dync1h1 | Cytoplasmic dynein 1 heavy chain 1 | 2.0 | 0.4 | **O** |  |  |
| IPI00130102 | Des | Desmin | 7.2 | 0.3 |  |  |  |
| IPI00322209 | Krt8 | Keratin, type II cytoskeletal 8 | 2.0 | 0.3 | **O** |  |  |
| IPI00851023 | Macf1 | Microtubule-actin crosslinking factor 1 | 0.2 | 0.1 |  |  |  |
| IPI00757353 | Spna2 | Spectrin alpha 2 | 2.0 | 0.4 |  |  |  |
| IPI00929758 | Tln1 | Talin 1 | 2.0 | 0.4 | **O** |  |  |
| IPI00320217 | Cct2 | T-complex protein 1 subunit beta | 2.0 | 0.3 | **O** | **O** | **O** |
| IPI00125778 | Tagln2 | Transgelin 2 | 4.0 | 0.4 | **O** |  |  |
| IPI00112251 | Tubb3 | Tubulin beta-3 chain | 11.9 | 0.4 | **O** | **O** | **O** |
|  |  | **Enzyme** |  |  |  |  |  |
| IPI00310035 | Fah | Fumarylacetoacetase | 2.0 | 0.1 | **O** |  | **O** |
| IPI00649103 | Gaa | Glucosidase, alpha, acid | 0.4 | 0.2 | **O** |  | **O** |
| IPI00111981 | Ola1 | Isoform 1 of Obg-like ATPase 1 | 0.5 | 0.2 | **O** |  |  |
| IPI00671847 | Ppp1r12a | Isoform 1 of Protein phosphatase 1 regulatory subunit 12A | 0.2 | 0.1 | **O** |  |  |
| IPI00129105 | Sae1 | Isoform 1 of SUMO-activating enzyme subunit 1 | 5.9 | 0.3 |  |  |  |
| IPI00130627 | Lgmn | Legumain | 0.5 | 0.2 |  |  |  |
| IPI00555140 | Pgm1 | Phosphoglucomutase 1 | 4.0 | 0.4 | **O** |  |  |
| IPI00457898 | Pgam1 | Phosphoglycerate mutase 1 | 3.1 | 0.3 | **O** |  |  |
|  |  | **Heme and Pentose phosphate metabolism** | |  |  |  |  |
| IPI00466919 | Pgd | Phosphogluconate dehydrogenase, decarboxylating | 2.0 | 0.4 | **O** |  |  |
| IPI00112719 | Alad | Delta-aminolevulinic acid dehydratase | 0.1 | 0.1 | **O** |  |  |
|  |  | **Inflammatory response** |  |  |  |  |  |
| IPI00128249 | Ahsg | Alpha-2-HS-glycoprotein | 2.0 | 0.3 | **O** |  |  |
| IPI00407657 | C8a | Complement component 8, alpha polypeptide | 0.2 | 0.1 |  |  |  |
| IPI00118437 | C8g | Complement component 8, gamma subunit, isoform CRA_b | 10.0 | 1.4 |  |  |  |
| IPI00928163 | Cfb | Complement factor B isoform 1 | 4.3 | 0.1 | **O** |  |  |
| IPI00321666 | H2-Q10 | H-2 class I histocompatibility antigen, Q10 alpha chain | 2.0 | 0.4 | **O** |  |  |
| IPI00121274 | C8b | Isoform 1 of Complement component C8 beta chain | 2.0 | 0.4 |  |  |  |
| IPI00117910 | Prx1 | Peroxiredoxin 1 | 6.0 | 0.3 | **O** | **O** |  |
| IPI00135547 | Saa4 | Serum amyloid A-4 protein | 23.7 | 0.2 | **O** |  |  |
| IPI00309214 | Apcs | Serum amyloid P-component | 14.8 | 0.4 | **O** |  |  |
| IPI00118413 | Thbs1 | Thrombospondin 1 | 3.7 | 0.3 | **O** |  |  |
|  |  | **Lipid metabolism** |  |  |  |  |  |
| IPI00226430 | Acaa2 | 3-ketoacyl-CoA thiolase, mitochondrial | 4.6 | 0.1 | **O** | **O** | **O** |
| IPI00126625 | Acsm1 | Isoform 1 of Acyl-coenzyme A synthetase ACSM1, mitochondrial | 3.1 | 0.3 | **O** |  |  |
| IPI00119114 | Acadl | Long-chain specific acyl-CoA dehydrogenase, mitochondrial | 2.0 | 0.4 | **O** |  | **O** |
| IPI00880477 | Slc27a5 | Slc27a5 46 kDa protein | 2.0 | 0.1 |  |  |  |
|  |  | **Membrane protein** |  |  |  |  |  |
| IPI00221652 | Prrg4 | Transmembrane gamma-carboxyglutamic acid protein 4 | 13.6 | 0.4 |  |  |  |
| IPI00127841 | Slc25a5 | ADP/ATP translocase 2 | 0.3 | 0.2 | **O** |  |  |
| IPI00117829 | Cav1 | Isoform Alpha of Caveolin 1 | 16.7 | 0.2 | **O** |  |  |
|  |  | **Oxidoreductase** |  |  |  |  |  |
| IPI00230084 | Aldh7a1 | Aldehyde dehydrogenase 7 family, member A1 isoform a | 0.1 | 0.1 | **O** | **O** | **O** |
| IPI00273096 | Akr1b8 | Aldose reductase-related protein 2 | 0.3 | 0.2 | **O** |  |  |
| IPI00117831 | Cp | Ceruloplasmin | 2.2 | 0.3 | **O** |  |  |
| IPI00331692 | Dci | Dodecenoyl-Coenzyme A delta isomerase precursor | 0.1 | 0.1 |  |  |  |
| IPI00128209 | Ak1 | Isoform 1 of Adenylate kinase isoenzyme 1 | 2.0 | 0.3 | **O** |  |  |
|  |  | **Protein folding** |  |  |  |  |  |
| IPI00123639 | Calr | Calreticulin | 2.1 | 0.1 | **O** | **O** |  |
| IPI00129526 | Hsp90b1 | Endoplasmin | 9.7 | 0.4 | **O** |  | **O** |
| IPI00469392 | Rtn4 | Isoform 1 of Reticulon 4 | 0.5 | 0.2 | **O** |  |  |
| IPI00331497 | Sept | Isoform 2 of Septin | 0.3 | 0.2 | **O** |  |  |
| IPI00114733 | Serpinh1 | Serpin H1 | 0.1 | 0.1 |  |  |  |
| IPI00136984 | Rps7 | 40S ribosomal protein S7 | 16.2 | 0.2 | **O** |  |  |
| IPI00122862 | Mthfd1 | C-1-tetrahydrofolate synthase, cytoplasmic | 2.2 | 0.3 | **O** | **O** | **O** |
| IPI00118825 | Csl | Citrate synthase-like protein | 0.1 | 0.1 |  | **O** |  |
| IPI00230355 | Gspt1 | G1 to S phase transition 1 isoform 1 | 0.3 | 0.2 | **O** |  |  |
| IPI00119095 | Gm2a | Ganglioside GM2 activator | 0.2 | 0.1 | **O** |  |  |
| IPI00153743 | Sfrs7 | Isoform 2 of Splicing factor, arginine/serine-rich 7 | 2.8 | 0.1 |  |  |  |
| IPI00673513 | Gm5045 | Similar to 60S ribosomal protein L7 isoform 2 | 0.1 | 0.1 |  | **O** | **O** |
| IPI00754157 | RPL3 | Similar to ribosomal protein L3 isoform 3 | 0.3 | 0.2 | **O** |  |  |
|  |  | **Proteolysis** |  |  |  |  |  |
| IPI00677690 | LOC675521 | Similar to complement component 4, gene 1 isoform 5 | 2.9 | 0.3 | **O** |  |  |
| IPI00133206 | Psmc3 | 26S protease regulatory subunit 6A | 0.2 | 0.1 | **O** |  |  |
| IPI00319509 | Anpep | Aminopeptidase N | 0.2 | 0.2 | **O** | **O** |  |
| IPI00331394 | Dnpep | Aspartyl aminopeptidase isoform A | 0.4 | 0.2 | **O** |  |  |
| IPI00955390 | C4 | C4a protein | 2.1 | 0.1 | **O** |  |  |
| IPI00759878 | C3 | Complement component 3 | 4.6 | 0.1 | **O** |  |  |
| IPI00381881 | C7 | Complement component 7 | 0.1 | 0.1 |  |  |  |
| IPI00320675 | Cfi | Complement factor I | 2.0 | 0.4 | **O** |  |  |
| IPI00224152 | Apex1 | DNA-(apurinic or apyrimidinic site) lyase | 2.0 | 0.4 | **O** |  |  |
| IPI00170126 | Pitrm1 | Isoform 1 of Presequence protease | 2.0 | 0.3 |  |  |  |
| IPI00319518 | Lonp1 | Lon protease homolog | 0.5 | 0.2 |  |  |  |
| IPI00130391 | Prss1 | Protease, serine, 1 | 2.9 | 0.1 |  |  |  |
| IPI00135635 | Serpina3m | Serine protease inhibitor A3M | 2.1 | 0.1 |  |  |  |
| IPI00314141 | Serpina3n | Serine protease inhibitor A3N | 2.0 | 0.4 |  |  |  |
|  |  | **Secreted protein** |  |  |  |  |  |
| IPI00121209 | Apoa1 | Apolipoprotein A-I | 3.5 | 0.3 | **O** |  | **O** |
| IPI00553333 | Hbb-b1 | Hemoglobin subunit beta-1 | 40.5 | 1.2 | **O** |  | **O** |
| IPI00464400 | Ig22 | Ig kappa chain V-III region ABPC 22/PC 9245 | 0.1 | 0.1 |  |  |  |
| IPI00138175 | Ig7175 | Ig kappa chain V-III region PC 7175 | 0.1 | 0.1 |  |  |  |
| IPI00624663 | Pzp | Alpha-2-macroglobulin | 3.9 | 0.4 | **O** |  |  |
| IPI00608020 | Ftl1 | Ferritin light chain 1 | 0.5 | 0.1 | **O** |  |  |
| IPI00409148 | Hp | Haptoglobin | 3.0 | 0.2 | **O** | **O** |  |
| IPI00128484 | Hpx | Hemopexin | 5.7 | 0.3 | **O** |  |  |
| IPI00177214 | Igh-6 | Ig mu chain C region membrane-bound form | 2.9 | 0.3 |  |  |  |
| IPI00138192 | Ig | Kappa chain V-VI region SAPC 10 | 2.7 | 0.4 | **O** |  |  |
| IPI00114206 | F2 | Prothrombin (Fragment) | 2.7 | 0.1 | **O** | **O** |  |
| IPI00131695 | Alb | Serum albumin | 3.4 | 0.3 | **O** | **O** | **O** |
| IPI00111315 | Apoa2 | Apolipoprotein A-II | 2.4 | 0.4 | **O** |  |  |
| IPI00123194 | Bgn | Biglycan | 0.5 | 0.2 | **O** |  | **O** |
| IPI00130654 | Afm | Isoform 3 of Afamin | 0.1 | 0.1 | **O** |  |  |
| IPI00322936 | Plg | Plasminogen | 2.4 | 0.4 | **O** |  |  |
|  |  | **Small GTPases** |  |  |  |  |  |
| IPI00230704 | Arhgef7 | Rho guanine nucleotide exchange factor 7, Isoform C | 2.1 | 0.4 |  |  |  |
| IPI00467447 | Iqgap1 | Ras GTPase-activating-like protein IQGAP1 | 10.0 | 0.4 | **O** |  |  |
| IPI00127408 | Rac1 | RAS-related C3 botulinum substrate 1, isoform CRA_A | 0.3 | 0.2 | **O** | **O** |  |
|  |  | **Transcription** |  |  |  |  |  |
| IPI00153400 | H2afj | Histone H2A.J | 6.1 | 0.3 | **O** | **O** | **O** |
| IPI00776156 | Smarca1 | SWI/SNF related, matrix associated, actin dependent regulator of chromatin, subfamily a, member 1 | 0.4 | 0.2 |  |  |  |
|  |  | **Transferase and binding protein** |  |  |  |  |  |
| IPI00466399 | Mup2 | Major urinary protein 2 precursor | 21.6 | 0.2 |  |  |  |
| IPI00322869 | Abce1 | ATP-binding cassette sub-family E member 1 | 2.6 | 0.4 | **O** |  |  |
| IPI00623845 | Selenbp1 | Selenium-binding protein 1 | 2.0 | 0.3 | **O** |  | **O** |
| IPI00604945 | Mpst | Mercaptopyruvate sulfurtransferase | 0.1 | 0.1 | **O** |  |  |
| IPI00408664 | Atad5 | Isoform 1 of ATPase family AAA domain-containing protein 5 | 0.1 | 0.1 |  |  |  |
| IPI00113992 | Gmppb | Mannose-1-phosphate guanyltransferase beta | 0.3 | 0.2 | **O** |  |  |
| IPI00116105 | Serpina6 | Corticosteroid-binding globulin | 4.3 | 0.4 |  |  |  |
| IPI00408378 | Ywhaq | Isoform 1 of 14-3-3 protein theta | 2.2 | 0.3 | **O** |  | **O** |
| IPI00317289 | Dctn4 | Isoform 2 of dynactin subunit 4 | 0.5 | 0.1 |  |  |  |
| IPI00129178 | Oat | Ornithine aminotransferase, mitochondrial | 0.5 | 0.1 | **O** |  |  |
| IPI00128904 | Pcbp1 | Poly(rC)-binding protein 1 | 5.8 | 0.3 | **O** |  |  |
| IPI00224740 | Pfn1 | Profilin 1 | 3.2 | 0.3 | **O** |  |  |
| IPI00119202 | S100a11 | Protein S100-A11 | 0.1 | 0.1 | **O** |  |  |
| IPI00126184 | Gc | Vitamin D-binding protein | 2.7 | 0.4 |  |  | **O** |
|  |  | **Xenobiotics/Endogenous compound metabolism** | | |  |  |  |
| IPI00221400 | Adh1 | Alcohol dehydrogenase 1 | 7.8 | 0.4 | **O** | **O** | **O** |
| IPI00228633 | Gpi1 | Glucose-6-phosphate isomerase | 129.6 | 12.0 | **O** |  |  |
| IPI00127691 | Gs | Glutathione synthetase | 66.0 | 1.6 | **O** |  |  |
| IPI00331241 | Gsta3 | Glutathione S-transferase A3 | 3.0 | 0.1 | **O** | **O** |  |
| IPI00131204 | Ugp2 | Isoform 1 of UTP-glucose-1-phosphate Uridylyltransferase | 0.3 | 0.2 | **O** |  |  |
| IPI00138342 | Ces1 | Liver carboxylesterase 1 | 4.4 | 0.1 |  |  | **O** |
| IPI00109142 | FGH | S-formylglutathione hydrolase | 2.0 | 0.4 | **O** |  | **O** |
| IPI00118344 | Ugdh | UDP-glucose 6-dehydrogenase | 0.1 | 0.1 | **O** | **O** | **O** |
|  |  | **Unknown** |  |  |  |  |  |
| IPI00115085 | Impa1 | Inositol monophosphatase 1 | 0.2 | 0.1 |  |  |  |
| IPI00886090 | Cct5 | Cct5 22 kDa protein | 0.5 | 0.2 | **O** |  |  |
| IPI00130530 | Grhpr | Glyoxylate reductase/hydroxypyruvate reductase | 3.9 | 0.4 | **O** |  |  |
| IPI00463297 | Gm5745 | Gm5745 hypothetical protein | 0.5 | 0.1 |  |  |  |
| IPI00265107 | Gm5044 | Hypothetical protein isoform 2 | 3.7 | 0.3 |  |  |  |
| IPI00319320 | Nckap1 | Isoform 1 of Nck-associated protein 1 | 2.3 | 0.4 | **O** |  |  |
| IPI00831432 | LOC544903 | LOC544903 Protein | 27.7 | 1.2 |  |  |  |
| IPI00330695 | Fam129b | Niban-like protein 1 | 2.0 | 0.3 | **O** |  |  |
| IPI00652813 | Fn1 | Putative uncharacterized protein | 24.2 | 1.2 | **O** |  |  |
| IPI00224151 | Ap1g1 | Adaptor-related protein complex 1, gamma 1 subunit | 2.2 | 0.3 | **O** |  |  |
| IPI00132089 | Prep | Prolyl endopeptidase | 2.0 | 0.4 | **O** |  |  |
| IPI00223437 | Copg | Coatomer protein complex, subunit gamma | 2.0 | 0.3 | **O** |  |  |
| IPI00653565 | Anxa3 | Annexin A3 | 0.1 | 0.1 | **O** |  |  |
| IPI00135150 | Kpna3 | Putative uncharacterized protein (Fragment) | 0.1 | 0.1 |  |  |  |
| IPI00355673 | Serpina3h | Serpina3h protein | 2.1 | 0.4 |  |  |  |

^a^Proteins from three independent biological replicate sets from CON- and APAP-derived EVs were used for gel-assisted digestion and then 2D-LC-MS/MS analysis. IDEAL-Q software was applied for the label-free quantitation strategy. The APAP EVs/control EVs ratio at protein level was measured by MS/MS analysis.

^b^Identified circulating EVs proteins were compared to the EVs compositions from ExoCarta.

^c^Identified circulating EVs proteins were compared to the exosomes composition from rat primary hepatocytes[[1](#_ENREF_1)].

^d^Identified circulating EVs proteins were compared to the EVs compositions from HepG2 cells.

**
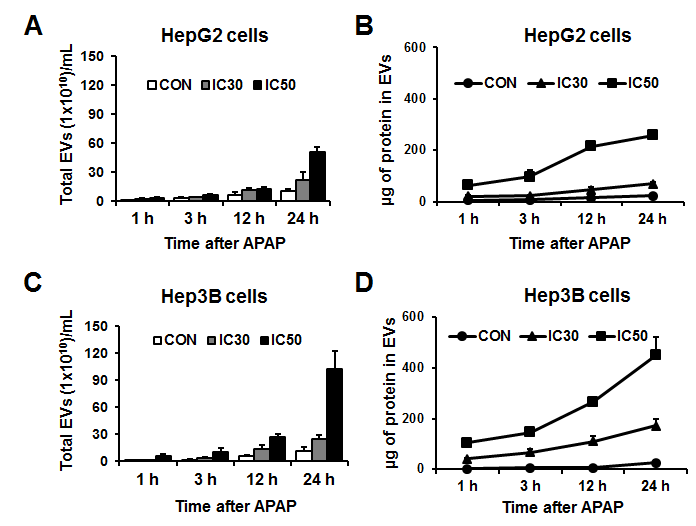
**

**Figure A in S1 File. The number and protein amount of EVs prepared from liver cell lines are increased by APAP.** HepG2 and Hep3B cells were treated with CON (growth media) or APAP at the IC_30_ dose, or APAP at the IC_50_ dose for 1, 3, 12, or 24 h, as indicated, and the EVs isolated from the culture supernatants (n = 3/group). (**A**) The numbers of EVs isolated from the HepG2 cell culture media were determined by NanoSight analysis. (**B**) The protein amounts in EVs, isolated from the HepG2 cell culture media, were quantified using protein analysis. (**C**) The numbers of EVs isolated from the Hep3B cell culture media were determined by NanoSight analysis. (**D**) The protein amounts in EVs, isolated from the Hep3B cell culture media, were quantified using protein analysis.


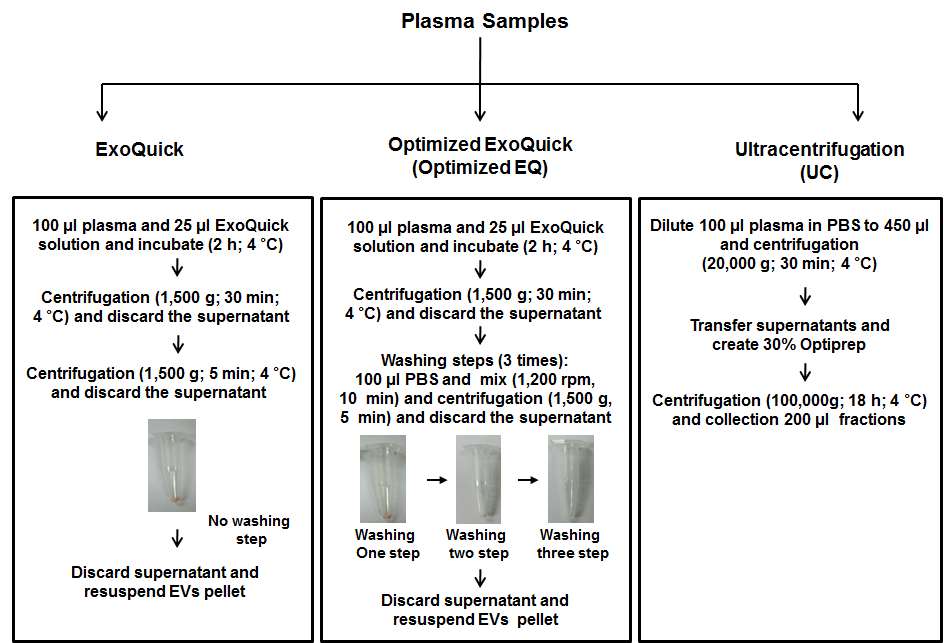


**Figure B in S1 File. Schematic overview of the 3 methods of EV isolation from mouse plasma.**

**
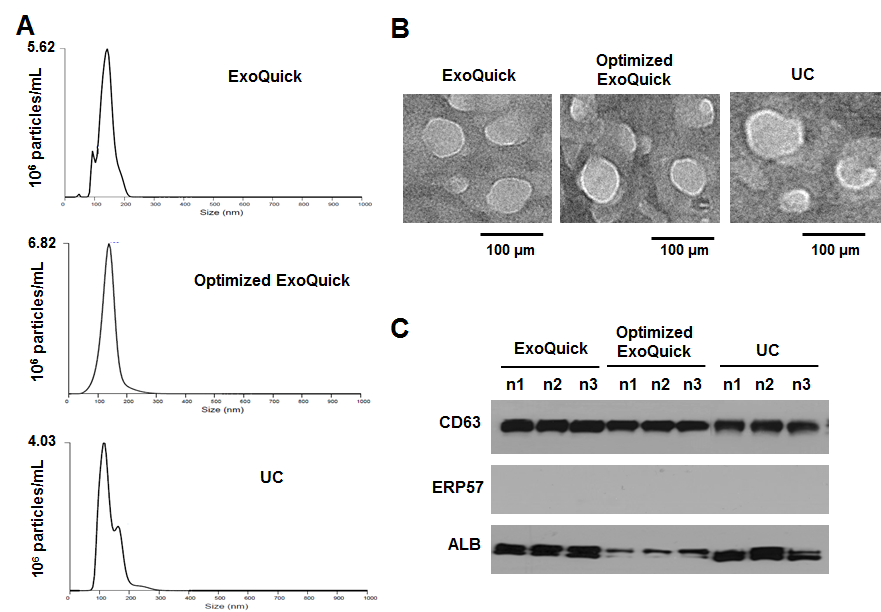
**

**Figure C in S1 File. Characteristics of plasma-derived EVs isolated by the three different methods.** EVs were isolated from plasma of control mice by the three different methods, as indicated. (**A**, **B**) The size profiles of EVs have been evaluated by NanoSight analysis (A) and TEM imaging (B). (**C**) Immunoblot analyses were performed with 20 μg proteins/well to determine the relative levels of CD63, ERP57, and albumin in plasma-derived EVs prepared by the three different methods.

**
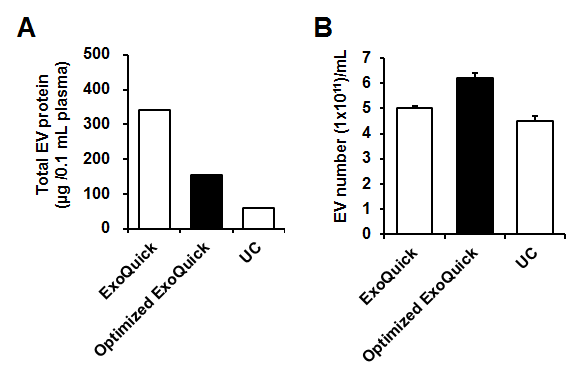
**

**Figure D in S1 File. Comparison of the number and total proteins in EVs isolated by ExoQuick, Optimized ExoQuick, and UC methods.** EVs were isolated from plasma of control mice by the indicated methods. (**A**) The protein amounts in EVs were isolated from the plasma by ExoqQuick, Optimized ExoQuick, and UC methods are presented (n = 3/group). (**B**) The numbers of EVs isolated from the plasma using the three methods were determined by NanoSight analysis (n = 3/group).

**
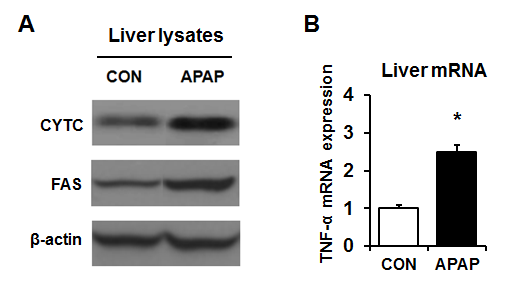
**

**Figure E in S1 File. APAP exposure induced liver injury in mice.** Wild-type male Balb/C mice (6 weeks old) were injected with a single ip dose of APAP (300 mg/kg, LD_50_ dosage) or saline (CON, negative control) for 24 h to produce APAP-induced hepatic injury. (**A, B**) The protein levels of cytochrome C (CYTC) and fatty acid synthase (FAS) (A) and mRNA level of TNF-α (B) in whole liver lysates were measured (n = 4/group), as indicated. Data represent the mean ± SD. **P* < 0.05.

**
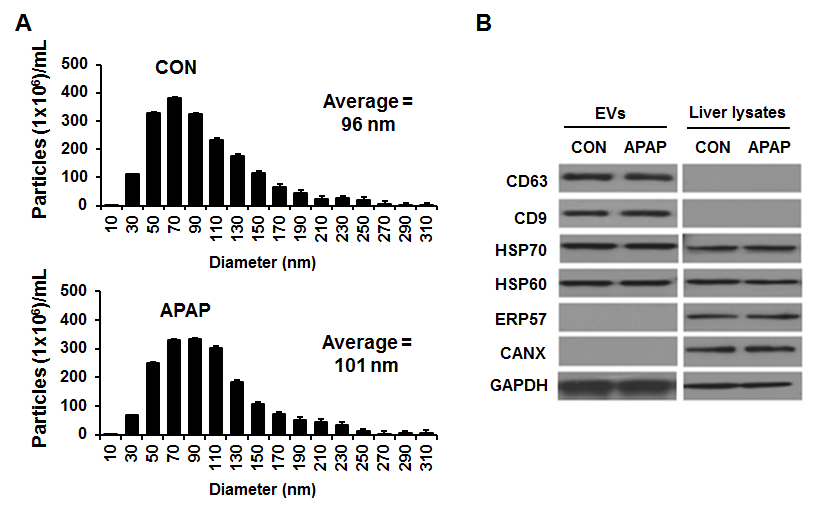
**

**Figure F in S1 File. Characteristics of circulating EVs from mouse plasma.** Wild-type male Balb/C mice (6 weeks old) were injected with a single ip dose of saline (CON) or APAP at 300 mg/kg for 24 h. (**A**) The number and size (nm) distribution of EVs isolated from plasma in CON and APAP-treated mice were determined by Nanoparticle Tracking Analysis (NanoSight). (**B**) Representative western blot analyses for the indicated proteins in circulating EVs and whole liver lysates. Endoplasmic markers, endoplasmic reticulum protein 57 (ERP57) and calnexin (CANX), were not detected in circulating EVs, but detected in whole liver lysates.

**
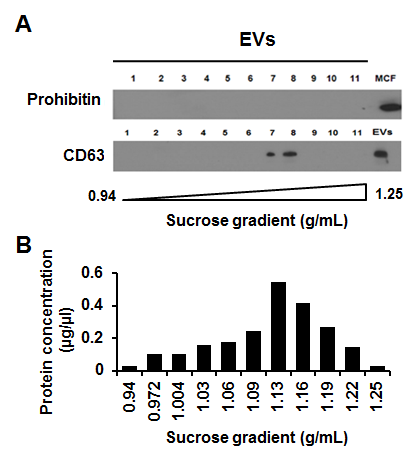
**

**Figure G in S1 File. Confirmation of EV marker protein in circulating EVs purified from mouse plasma on a discontinuous sucrose gradient.** (**A**, **B**) Plasma-derived circulating EVs from control mice were loaded on a discontinuous sucrose gradient (0.94–1.25 g/mL). Eleven fractions were collected. Immunoblot analysis were performed to verify the distribution of EV proteins on the sucrose gradient by detecting CD63 as a marker of EV protein. Immunoblot analysis revealed that prohibitin, an apoptosis bleb marker, was not associated with EV fractions, although it was detected in MCF cell lysates, used as a positive source.

**
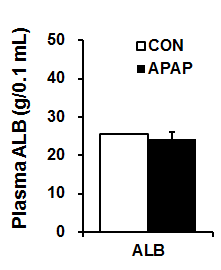
**

**Figure H in S1 File. The amount of mouse plasma ALB was not changed by APAP.**


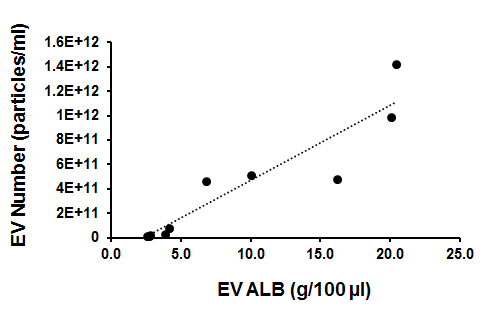


**Figure I in S1 File. The correlation of EV protein with EV ALB level was identified by correlation test.**

**
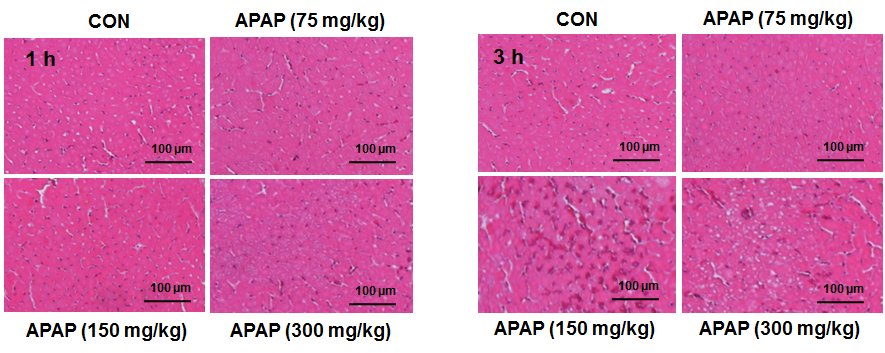
**

**Figure J in S1 File.APAP induces centrilobular hepatocellular necrosis in a dose- and time-dependent manner.** Male BALB/c mice (6 weeks old) were treated with a single ip injection of 75, 150, or 300 mg/kg APAP for 1 or 3 h (n = 10/group). Representative H&E stained slides of formalin-fixed liver sections of the indicated groups are presented. Scale bars, 100 µm.

**
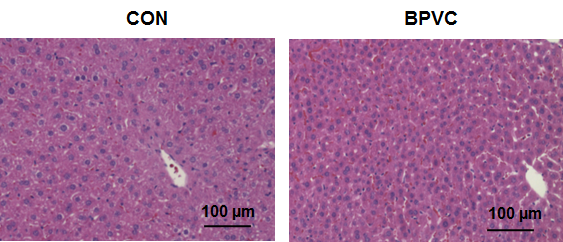
**

**Figure K in S1 File. Myotoxic BPVC does not induce centrilobular hepatocellular necrosis.** Male BALB/c mice (6 weeks old) were treated with 0 (CON) or 0.5% BPVC via intramuscular injection and sacrificed after 24 h. Representative H&E staining of formalin-fixed liver slides of the indicated groups are shown (n = 10/group). Scale bars, 100 µm.
